# Pluronic F-68 Improves Root Growth of Recalcitrant Rice Cultivar Through Enhanced Auxin Biosynthesis

**DOI:** 10.21315/tlsr2025.36.2.12

**Published:** 2025-07-31

**Authors:** Andrew De-Xian Kok, Janna Ong-Abdullah, Amanda Shen-Yee Kong, Rogayah Sekeli, Chien-Yeong Wee, Swee-Hua Erin Lim, Wan-Hee Cheng, Jiun-Yan Loh, Kok-Song Lai

**Affiliations:** 1 Department of Cell and Molecular Biology, Faculty of Biotechnology and Biomolecular Sciences, Universiti Putra Malaysia, 43400 UPM Serdang, Selangor, Malaysia; 2 School of Pharmacy, Monash University Malaysia, Jalan Lagoon Selatan, Bandar Sunway, 47500 Subang Jaya, Selangor, Malaysia; 3 Biotechnology and Nanotechnology Research Centre, Malaysian Agricultural Research and Development Institute (MARDI), Kuala Lumpur, Malaysia; 4 Health Sciences Division, Abu Dhabi Women’s College, Higher Colleges of Technology, 25026 Abu Dhabi, United Arab Emirates; 5 Faculty of Health and Life Sciences, INTI International University, Persiaran Perdana BBN, Putra Nilai, 71800 Nilai, Negeri Sembilan, Malaysia; 6 Tropical Futures Institute, James Cook University Singapore, 149 Sims Drive 387380, Singapore

**Keywords:** Auxin Biosynthesis, Root Growth, Pluronic F-68, Recalcitrant *Indica* cv. MR, Biosintesis Auksin, Pertumbuhan Akar, Pluronik F-68, *Indica* Rekalsitran cv. MR 219

## Abstract

In plants, roots play a vital role in crop performance and yield that impact the agricultural productivity. Pluronic F-68 (PF-68) is a type of non-ionic surfactant that is typically utilised as a plant growth additive. There is a lack of studies on the impact of PF-68 on root growth. This work aims to assess the impacts of PF-68 on recalcitrant MR 219 rice root growth. Supplementation of 0.04% PF-68 enhanced the length (18.50%) and number of roots (15.87%) of MR 219 rice. The PF-68-treated MR 219 rice also showed a significant increment in sugar accumulation (1.73 mg/mL) and glutamate synthase activity (0.88 μmol/g protein). Consistent with the root growth enhancement, MR 219 rice supplemented with PF-68 recorded an increase in transcription levels of *Indole-3-Acetic Acid 23* (*OsIAA23*) (1.84-folds) and *WUSCHEL-Related Homeobox 11* (*OsWOX11*) (2.00-folds). Moreover, the PF-68-treated MR 219 rice also exhibited an enhancement of indole acetic acid (IAA) concentrations (27.33 ng/g FW), further suggesting its role in auxin biosynthesis. Taken together, our study revealed that the introduction of PF-68 enhanced the root growth of MR 219 rice through improved sugar accumulation, glutamate synthase activity and auxin biosynthesis.

## Abstrak

Dalam tumbuhan, akar memainkan peranan penting dalam prestasi tanaman dan hasil yang memberi kesan kepada produktiviti pertanian. Pluronic F-68 (PF-68) adalah sejenis surfaktan bukan ionik yang biasanya digunakan sebagai bahan tambahan pertumbuhan pokok. Tidak banyak kajian dibuat berkenaan kesan PF-68 terhadap pertumbuhan akar. Kajian ini bertujuan untuk menilai kesan PF-68 terhadap pertumbuhan akar padi MR 219 yang rekalsitran. Penambahan 0.04% PF-68 meningkatkan kepanjangan (18.50%) dan bilangan (15.87%) akar pokok padi MR 219. Pokok yang dirawat juga menunjukkan peningkatan yang ketara dalam pengumpulan gula (1.73 mg/mL) dan aktiviti sintase glutamat (0.88 μmol/g protein). Selaras dengan peningkatan pertumbuhan akar, pokok-pokok tersebut juga merekodkan peningkatan tahap transkripsi *Indole-3-Acetic Acid 23* (*OsIAA23*) (1.84 kali ganda) dan *WUSCHEL-Related Homeobox 11* (*OsWOX11*) (2.00 kali ganda). Tambahan pula, pokok MR 219 yang dirawat PF-68 juga menunjukkan peningkatan kepekatan asid indole asetik (IAA) (27.33 ng/g FW), seterusnya mencadangkan peranannya dalam biosintesis auksin. Secara keseluruhan, kajian kami mendedahkan bahawa pengenalan PF-68 meningkatkan pertumbuhan akar pokok MR 219 melalui peningkatan pengumpulan gula, aktiviti sintase glutamat dan biosintesis auksin.

**Highlights**

- The 0.04% PF-68 significantly increased both the length and number of roots in the recalcitrant MR 219 rice.
- PF-68 treatment of MR 219 rice resulted in elevated indole-3-acetic acid concentrations, providing further evidence of its role in auxin biosynthesis.
- PF-68 has the potential to stimulate root growth, thereby enhancing rice production.

## INTRODUCTION

Rice stands as the paramount staple for over half the global population. Its cultivation spans approximately 154 million hectares annually, primarily in Asia. Predictions by the United Nations initially suggested an 8-billion-person world by 2025 (Khush 2005). To meet the escalating demand driven by population growth, rice production needs a 40% boost (Low *et al.* 2018). However, this improvement must occur amid constraints like limited land, reduced water, labour and fertiliser usage (Coudert *et al.* 2010; Kok *et al*. 2018). Enhancing root growth becomes pivotal for amping up rice production. A larger root system facilitates greater soil access, crucial for water and nutrient absorption at varying depths (Meng *et al.* 2019).

As a non-ionic surfactant, Pluronic F-68 (PF-68) finds application in plant and animal cultures (Barbulescu *et al.* 2011; Meier *et al.* 1999). In animal cell cultures, PF-68 aids cell growth stimulation, protection and the repair of damaged cells in suspension (Meier *et al.* 1999). In plant studies, PF-68 has shown efficacy in enhancing multiple shoot regeneration in various species like *Pyrus communis* (Dashti *et al*. 2012), *Ricinus communis* L. (Kulathuran & Narayanasamy 2015), as well as *Abelmoschus esculentus* L. (Irshad *et al.* 2018). Furthermore, it has been reported to bolster root growth in *Solanum dulcamara* (Kumar *et al.* 1990) and *Corchorus capsularis* L. (Khatun *et al.* 1993). Recent studies have highlighted PF-68’s potential in promoting callus proliferation in recalcitrant indica rice (Kok *et al.* 2020; 2021). In addition, PF-68-treated callus also showed increased root formation, suggesting its potential role in stimulating rice root growth (Kok *et al.* 2020; 2021).

Although PF-68’s effects on plants have been extensively studied, its underlying mechanism and specific impact on root growth remain largely unexplored, especially concerning rice, a crucial global food crop. Moreover, a better understanding of the role of PF-68 in root growth will allow its use to enhance crop growth and improve food security. Therefore, this investigation aims to assess PF-68’s influence on the root growth of a challenging rice variety.

## MATERIALS AND METHODS

For this study, seeds from the Malaysian rice cultivar MR 219 were utilised. Analytical-grade PF-68 (10%) from Thermo Fisher Scientific, USA, was employed. Seed surface sterilisation followed a previously outlined procedure with minor adaptations (Lim & Lai 2017). In brief, mature seeds underwent de-husking and were surface-sterilised using 70% ethanol for 1 min, and then 50% Clorox for 30 min. Post-sterilisation, the seeds were rinsed with distilled water and air-dried. These sterilised seeds were cultured on shoot induction medium consisting of Gamborg’s B5 basal medium (Gamborg *et al.* 1968) supplemented with specific nutrients and hormones under controlled conditions (Low *et al.* 2019). After one week, approximately 1 cm of shoot apices were excised and cultured on a root growth medium containing Murashige and Skoog medium (Murashige & Skoog 1962) with varying PF-68 concentrations [0.02%, 0.04%, 0.06%, 0.08% and 0.10% (v/v)]. The medium without PF-68 [0% (v/v)] served as a control. The rooted shoot apices were then incubated under specific light and temperature conditions for three weeks, following which root length and number were recorded. Each treatment was replicated thrice with 10 samples per replicate (*n* = 10).

Moreover, approximately 0.5 g of root samples underwent soluble sugar measurement using the phenol-sulphuric acid method (Terzi *et al.* 2014), measuring absorbance at 565 nm. Additionally, glutamate synthase (GOGAT) activity was assessed following Ertani *et al.*’s (2011) method. Root samples (approximately 0.5 g) were ground into powder with liquid nitrogen for GOGAT activity analysis, measuring absorbance at 340 nm.

RNA isolation from plant sample powder subjected to different treatments (control and 0.04% PF-68) followed Lai and Masatsugu’s (2013) protocol using the RNeasy Plant Mini Kit (Qiagen, Germany) (Lai & Masatsugu 2013). For first-strand cDNA synthesis, 1 μg of extracted total RNA was processed with the QuantiNova Reverse Transcription Kit (Qiagen, Germany). The primers were designed using the Primer-Blast from the National Centre for Biotechnology Information (NCBI) (see Table S1 in Supplementary Material) and synthesised by Integrated DNA Technologies (IDT, USA). Real-time PCR was executed on a Bio-Rad CFX96 system (Bio-Rad, US) with QuantiNova SYBR Green PCR (Qiagen, Germany), following the methodology outlined by Lai *et al.* (2011). PCR conditions comprised an initial step at 95ºC for 30 s, followed by 40 cycles of 95ºC for 5 s and 60ºC for 5 s. Each sample underwent three technical replicates across three biological replicates. Data analysis was carried out using Bio-Rad CFX Manager 3.1 software, and relative expression levels (2-ΔΔCT) were calculated using Livak’s method (Livak & Schmittgen 2001). The *rice cyclophilin* (*OsCYC*) and *ubiquitin 5* (*OsUBQ5*) were employed as reference genes in this study.

To assess indole-3-acetic acid (IAA) levels in both control and 0.04% PF-68-treated roots, the method outlined by Pan *et al.* (2010) was followed. Analysis was conducted using Agilent 1100 HPLC (Agilent Technologies, United States), and IAA levels were quantified using an external standard method (ng/g FW) with three biological replicates (Pan *et al.* 2010). All data presented are the mean ± standard error of the mean (SEM) from three biological replicates, each with three technical replicates. Statistical analysis, conducted using one-way analysis of variance (*p* < 0.05) between treatments, was performed utilising the IBM Statistical Package for the Social Sciences version 20.0.

## RESULT AND DISCUSSIONS

**Figure 1:**
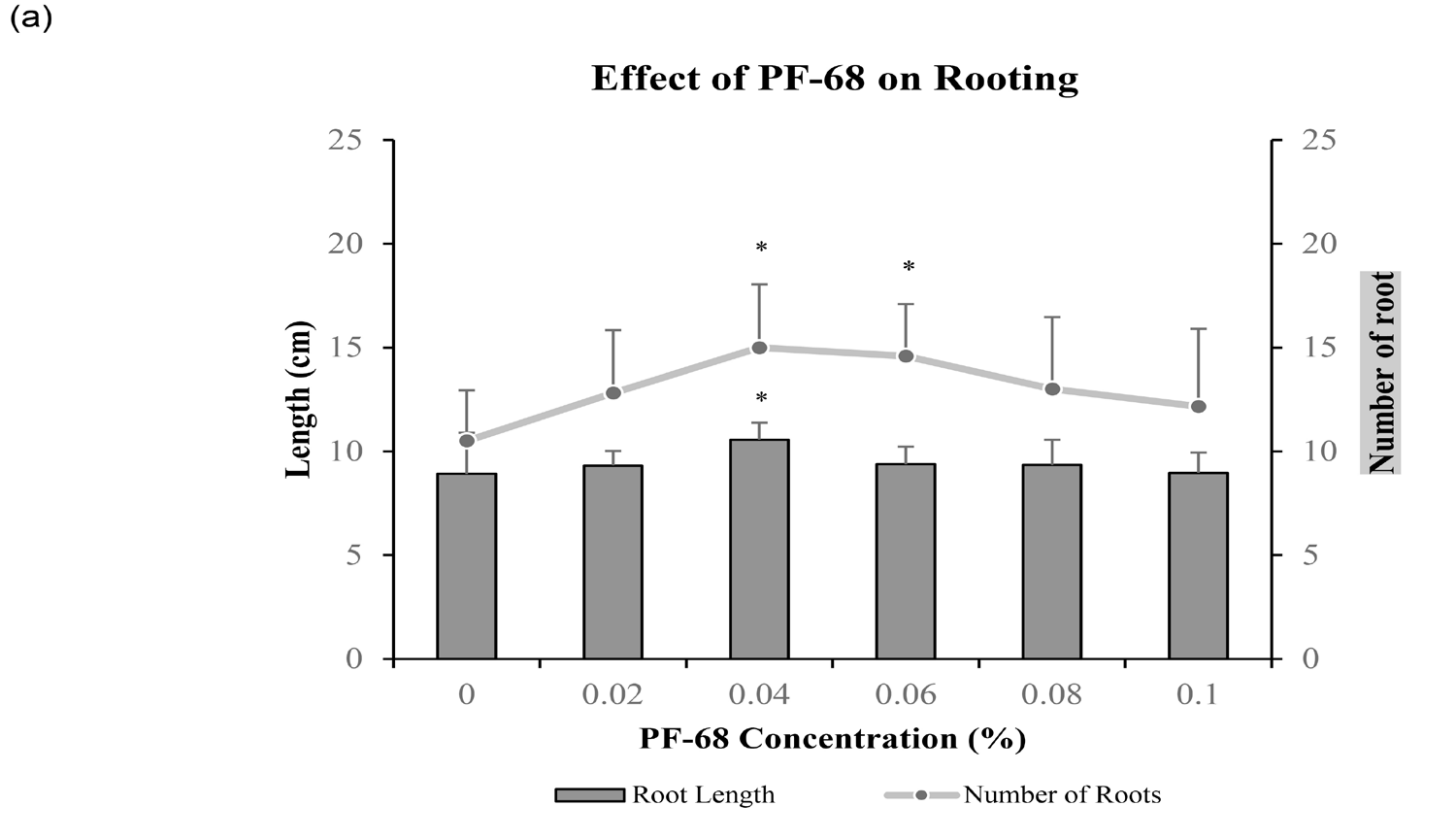

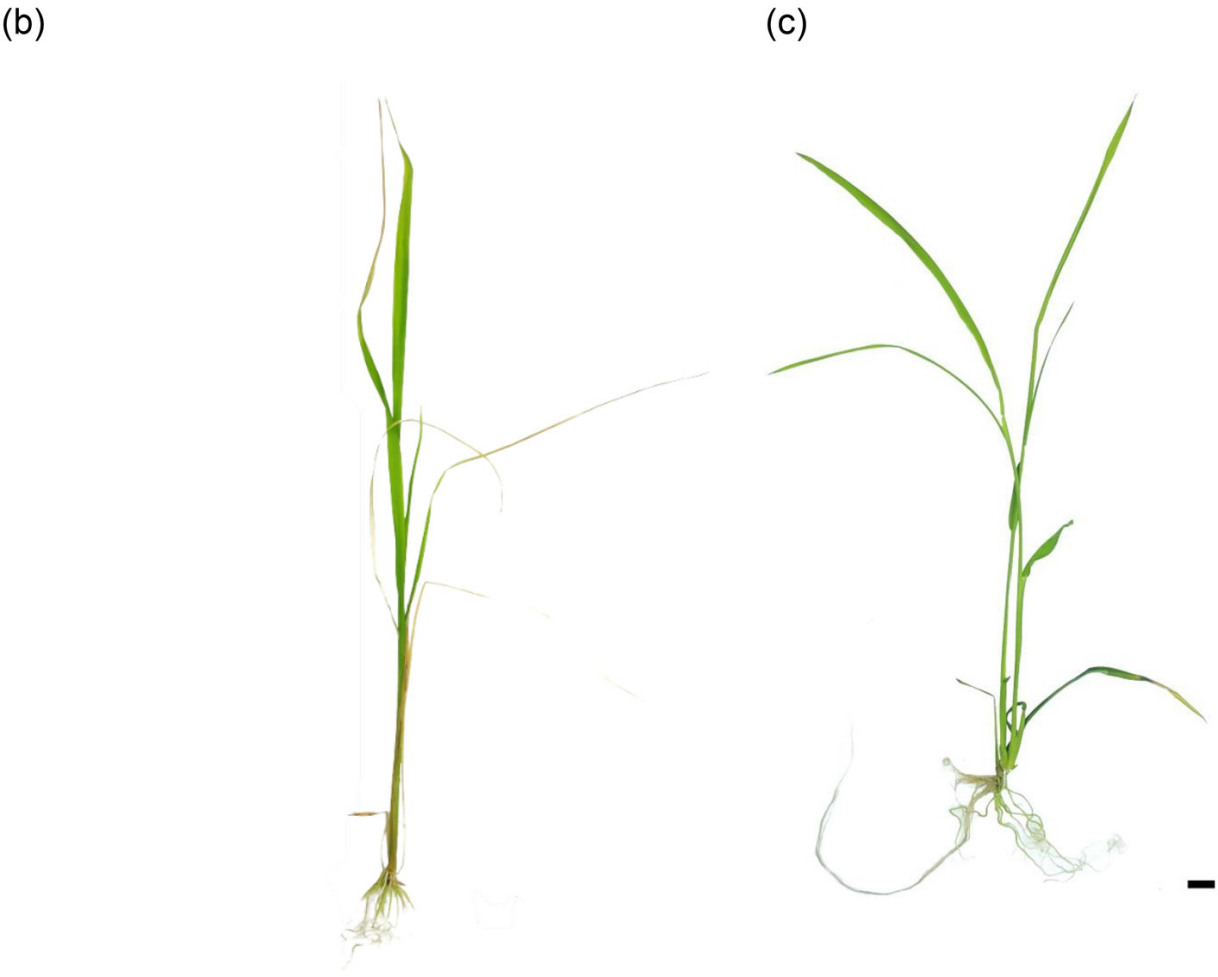
Effects of different concentrations of PF-68 on root growth. (a) Root length and number of roots recorded after four weeks of incubation. Morphology of MR 219 shoot apex grown on (b) control and (c) medium supplemented with 0.04% PF-68. Data shows mean of three biological replicates (*n* = 10). Asterisks indicate statistical significance difference at *p* < 0.05 compared to control. Error bars represent standard error mean. Scale bar = 0.5 cm.

This research showcases the efficacy of PF-68 in augmenting root growth in MR 219 rice (Fig. 1). Specifically, the addition of 0.04% and 0.06% PF-68 significantly bolstered (*p* < 0.05) root count by 42.85% and 38.89%, respectively. Conversely, 0.10% PF-68 exhibited the least stimulating effect on root production (15.87%) (Fig. 1a). Moreover, 0.04% PF-68 significantly enhanced (*p* < 0.05) root length by 18.50% (Fig. 1a), while 0.10% PF-68 displayed minimal impact on root length (0.55%) (Fig. 1a). Notably, the lower concentration (0.04%) of PF-68 demonstrated more favourable effects on root growth compared to the higher concentration (0.10%) (Fig. 1a).

**Figure 2:**
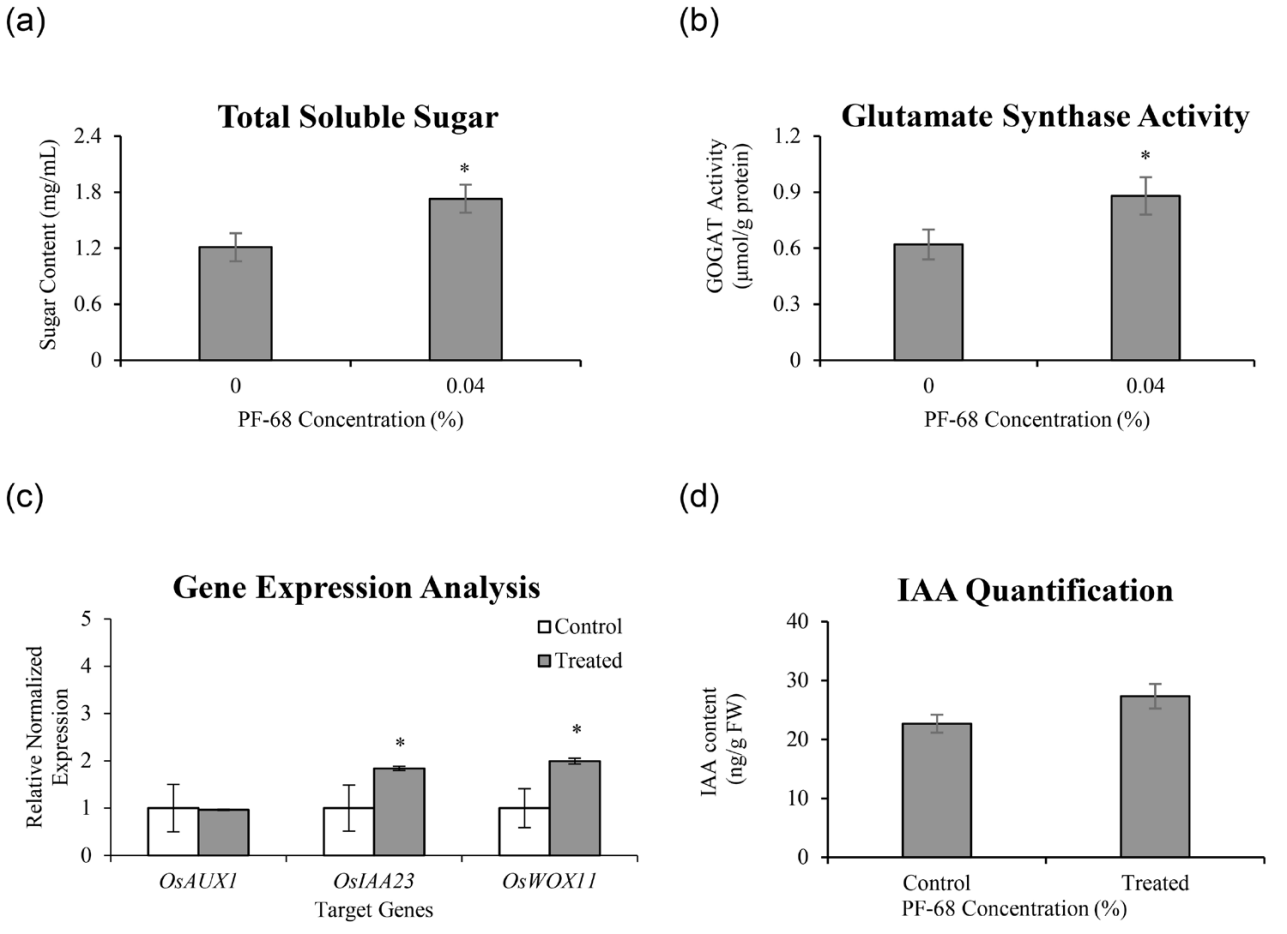
Biochemical assays, gene expression analysis and indole acetic acid (IAA quantification performed on control and 0.04% PF-68 treated roots. (a) Total sugar content, (b) GOGAT activity, (c) Normalised relative gene expression of selected genes (OsAUX1, OsIAA23 and OsWOX11) and (d) IAA quantification. *Notes.* Data shows the mean of three biological replicates. Asterisk indicates statistically significant at *p* < 0.05 compared to control (0% PF-68). Error bars represent standard error mean.

Biochemical assessments on control and 0.04% PF-68-treated rice explants revealed a substantial increase in total sugar content (1.73 mg/mL) and GOGAT activity (0.88 μmol/g protein) in the treated MR 219 rice compared to the control (Figs. 2a and 2b). This suggests that the presence of PF-68 promotes root growth via heightened sugar accumulation and GOGAT activity. Further gene expression analysis via real-time PCR focused on three target genes involved in auxin biosynthesis (Fig. 2c). Notably, 0.04% PF-68 treatment resulted in significant increments of *WUSCHEL-Related Homeobox 11* (*OsWOX11*) (2.00-folds) and *indole-3-acetic acid 23* (*OsIAA23*) (1.84-folds) transcripts compared to the control. Additionally, IAA quantification revealed higher content in 0.04% PF-68-treated rice explants (27.33 ± 2.08 ng/g FW) versus the control (22.67 ± 1.53 ng/g FW) (Fig. 2d), indicating PF-68’s potential role in auxin biosynthesis. While these results may not be statistically significant, they could be due to limitations in sample size and uncontrollable variability. However, it’s important to note that statistical significance does not necessarily imply a lack of effect. Hence, we could not completely rule out the positive effect that PF-68 has on the IAA.

Root development significantly impacts plant growth and nutrient absorption. This study demonstrated that 0.04% PF-68 supplementation effectively enhanced MR 219 rice root growth (Fig. 1). Considering the fixed external nutrient supply in each treatment, this suggests that PF-68 might facilitate root growth in MR 219 by improving nutrient acquisition. Earlier reports also align with our findings, indicating PF-68’s ability to enhance root growth at lower concentrations, with diminishing effects as the concentration increases (Kumar *et al.* 1990).

Soluble sugars play a pivotal role in plant functions, influencing metabolism, growth and development. For instance, Eveland and Jackson (2011) highlighted how sugar accumulation fosters cell growth by fuelling carbon and energy production through carbohydrate metabolism (Eveland & Jackson 2011). Both carbon and nitrogen assimilation are vital for overall plant development and are intricately interconnected (Jiang *et al.* 2024). Studies have shown that a decrease in soluble sugar content significantly hampers protein synthesis and diminishes nitrogen utilisation efficiency in plants (Hao *et al.* 2020). This reduction in carbon and nitrogen metabolism affects amino acid biosynthesis, crucial as nitrogen serves as a fundamental building block for amino acids (Lehmeier *et al.* 2013). GOGAT, a key player in nitrogen metabolism, exhibited heightened activity in 0.04% PF-68-treated explants, suggesting an enhancement in nitrogen metabolism. The observed increase in soluble sugar accumulation and GOGAT activity (Fig. 2) in these explants indicates PF-68’s pivotal role in promoting root growth through carbohydrate and nitrogen metabolisms.

Multiple genes associated with plant root growth, including *OsAUX1*, *OsIAA23* and *OsWOX11*, underwent scrutiny via gene expression analysis. OsAUX1 manages auxin transport, pivotal for root growth and lateral root development (Péret *et al.* 2012; Wiśniewska *et al.* 2024). It also facilitates the accumulation of IAA in the root apex, aiding lateral root development (Wiśniewska *et al.* 2024). Mutations in AUX1 have revealed developmental issues linked to auxin, like extended primary roots and shortened root hairs in rice (Yu *et al.* 2015). Agravitropic roots and diminished lateral root initiation have been observed in the mutant OsAUX1 (Swarup *et al*. 2005; Zhao *et al.* 2015). Nonetheless, scrutiny of OsAUX1 transcript in 0.04% PF-68-treated samples (Fig. 2d) indicated no alterations, suggesting that PF-68 application does not influence AUX1, the auxin transporter’s regulation.

In plant, the importance of auxin biosynthesis in root development has been extensively documented. IAA, the primary natural auxin, chiefly originates from tryptophan via Trp-dependent and Trp-independent pathways (Zhao *et al.* 2015). Various studies have highlighted the impact of hindering Trp production on root development (Nishimura *et al.* 2014; Soeno *et al.* 2010). For instance, inhibiting Trp aminotransferase with L-amino-oxyphenylpropionic acid (AOPP) in Arabidopsis led to deficiencies in root growth and development (Soeno *et al.* 2010). Similarly, moderate aluminium supply to tea plant roots significantly boosted their IAA content, resulting in notable improvements in lateral root number and length (Gao *et al.* 2022). In this study, the increase in IAA content (Fig. 2d) in 0.04% PF-68-treated samples aligned with the rise in both root number and length (Fig. 1).

Crown roots are pivotal components of the fibrous root system in rice (Zhao *et al.* 2015). Genes such as *IAA23* and *WOX11* contribute to crown root development in plants (Islam *et al.* 2021; Jun *et al*. 2011). *IAA23* expression is specific to quiescent centre cells during various root developments (Meng *et al.* 2019). Loss of OsIAA23 function in rice resulted in root cap disintegration and halted root growth (Jun *et al.* 2011). *WOX11* expression in early crown root primordia and the root meristem’s cell division zone play crucial roles (Coudert *et al.* 2010). Zhao *et al.* (2009) demonstrated that auxin treatment failed to induce crown root production in rice wox11 mutants, while overexpressing *WOX11* led to early crown root growth and increased root biomass (Zhao *et al.* 2009). Enhanced expression of OsIAA23 and OsWOX11 transcripts in 0.04% PF-68-treated samples (Fig. 2c) corresponded with increased root number (Fig. 1a). These results support PF-68’s capacity to enhance root growth by regulating OsIAA23 and OsWOX11 transcripts.

## CONCLUSION

In summary, PF-68 application significantly bolstered root growth in the MR 219 cultivar. Enhanced carbon and nitrogen metabolism in 0.04% PF-68-treated samples, indicated by carbohydrate accumulation and increased GOGAT activity, were evident. Furthermore, upregulated genes involved in crown root development and elevated IAA content suggest increased auxin biosynthesis in 0.04% PF-68- treated samples. Overall, PF-68’s efficacy is concentration-dependent, making it a valuable plant supplement for stimulating root growth.

## SUPPLEMENTARY MATERIAL

**Table S1:** Primers used for respective genes.

Target genes	Primer sequence 5′–3′	
*OsAUX1*	Forward	GGTAGAAGAAGAAGAGGGC
	Reverse	CCAAACAAACACAAGGACA
*OsIAA23*	Forward	GATCCTACCACACGCAACGA
	Reverse	GCCGTCGTCCAAAAACCAAA
OsWOX11	Forward	CCAGATGGGCGAGAGCTACT
	Reverse	CGTTGCCATCGATCAATCAA

Housekeeping genes	Primer sequence 5′–3′	

*OsCYC*	Forward	GGTGTCACTCATGACTTCTG
	Reverse	GCCCATCCGAAACGATAC
*OsUBQ5*	Forward	TAGGCGTAGGCTCCTGTTCT
	Reverse	ACAGAGGTGATGCTAAGGTGT

## References

[b1-tlsr-36-2-253] Barbulescu DM, Burton W, Salisbury P (2011). Pluronic F-68: An answer for shoot regeneration recalcitrance in microspore-derived Brassica napus embryos. In Vitro Cellular & Developmental Biology – Plant.

[b2-tlsr-36-2-253] Coudert Y, Périn C, Courtois B, Khong NG, Gantet P (2010). Genetic control of root development in rice, the model cereal. Trends in Plant Science.

[b3-tlsr-36-2-253] Dashti SAHAV, Abdollahi H, Chamani M, Dashti (2012). Effects of Pluronic F-68 on regeneration and rooting of two pear cultivars (*Pyrus communis* cvs Dar Gazi and Bartlett). International Research Journal of Applied and Basic Sciences.

[b4-tlsr-36-2-253] Ertani A, Francioso O, Tugnoli V, Righi V, Nardi S (2011). Effect of commercial lignosulfonate-humate on *Zea mays* L. metabolism. Journal of Agricultural and Food Chemistry.

[b5-tlsr-36-2-253] Eveland AL, Jackson DP (2011). Sugars, signalling, and plant development. Journal of Experimental Botany.

[b6-tlsr-36-2-253] Gamborg OL, Miller RA, Ojima K (1968). Nutrient requirements of suspension cultures of soybean root cells. Experimental Cell Research.

[b7-tlsr-36-2-253] Gao Y, Wang M, Shi Y, Yang L, Hu J, Fan K, Shi Y (2022). IAA accumulation promotes the root growth of tea plants under aluminum. Agronomy.

[b8-tlsr-36-2-253] Hao J, li Q, Yu H, Wang H, Chai L, Miao T, Jiang W (2020). Comparative proteomic analysis of cucumber fruits under nitrogen deficiency at the fruiting stage. Horticultural Plant Journal.

[b9-tlsr-36-2-253] Irshad M, Rizwan D-H, Debnath B, Li M, Anwar M, Liu S, He B, Qiu D (2018). Ascorbic acid controls lethal browning and Pluronic F-68 promotes high-frequency multiple shoot regeneration from cotyldonary node explant of okra (*Abelmoschus esculentus* L.). HortScience: A publication of the American Society for Horticultural Science.

[b10-tlsr-36-2-253] Islam A, Zhang Y, Anis G, Rani M, Zegeye W, Yang Q, Liu L, Shen X, Cao L, Cheng S, Wu W (2021). Fine mapping and candidate gene analysis of qRN5a, a novel QTL promoting root number in rice under low potassium. Theoretical and Applied Genetics.

[b11-tlsr-36-2-253] Jiang Z, Chen Q, Liu D, Tao W, Gao S, Li J, Lin C, Zhu M, Ding Y, Li W, Li G, Sakr S, Xue L (2024). Application of slow-controlled release fertilizer coordinates the carbon flow in carbon-nitrogen metabolism to effect rice quality. BMC Plant Biology.

[b12-tlsr-36-2-253] Jun N, Gaohang W, Zhenxing Z, Huanhuan Z, Yunrong W, Ping W (2011). OsIAA23- mediated auxin signaling defines postembryonic maintenance of QC in rice. Plant Journal.

[b13-tlsr-36-2-253] Khatun A, Laouar L, Davey MR, Power JB, Mulligan BJ, Lowe KC (1993). Effects of Pluronic F-68 on shoot regeneration from cultured jute cotyledons and on growth of transformed roots. Plant Cell, Tissue and Organ Culture.

[b14-tlsr-36-2-253] Khush GS (2005). What it will take to Feed 5.0 Billion Rice consumers in 2030. Plant Molecular Biology.

[b15-tlsr-36-2-253] Kok A, Yoon L, Sekeli R, Wee C-Y, Balia Yusof ZN, Lai KS, Shah F, Khan ZH, Iqbal A (2018). Iron biofortification of rice: Progress and prospects. Rice crop: Current developments.

[b16-tlsr-36-2-253] Kok AD, Mohd Yusoff NF, Sekeli R, Wee CY, Lamasudin DU, Ong-Abdullah J, Lai KS (2021). Pluronic F-68 improves callus proliferation of recalcitrant rice cultivar via enhanced carbon and nitrogen metabolism and nutrients uptake. Frontiers in Plant Science.

[b17-tlsr-36-2-253] Kok AD, Wan Abdullah W, Tan NP, Ong-Abdullah J, Sekeli R, Wee CY, Lai KS (2020). Growth promoting effects of Pluronic F-68 on callus proliferation of recalcitrant rice cultivar. 3 Biotech.

[b18-tlsr-36-2-253] Kulathuran GK, Narayanasamy J (2015). Evaluation of Pluronic F68 and PGR’s for high frequency somatic embryogenesis and plant regeneration in castor (*Ricinus communis* L.) through solid culture. International Journal of Current Biotechnology.

[b19-tlsr-36-2-253] Kumar V, Laouar L, Davey MR, Mulligan BJ, Lowe KC (1990). Effects of Pluronic F-68 on growth of transformed roots of *Solanum dulcamara*. Biotechnology Letters.

[b20-tlsr-36-2-253] Lai K-S, Abdullah P, Yusoff K, Mahmood M (2011). An efficient protocol for particle bombardment-mediated transformation of Centella asiatica callus. Acta Physiologiae Plantarum.

[b21-tlsr-36-2-253] Lai KS, Masatsugu T (2013). Isolation and characterization of an arabidopsis thaliana self-incompatibility mutant induces by heavy-ion beam irradiation. Acta Biologica Cracoviensia s. Botanica.

[b22-tlsr-36-2-253] Lehmeier CA, Wild M, Schnyder H (2013). Nitrogen stress affects the turnover and size of nitrogen pools supplying leaf growth in a grass. Plant Physiology.

[b23-tlsr-36-2-253] Lim YY, Lai KS (2017). Generation of transgenic rice expressing cyclotide precursor *Oldenlandia affinis* kalata B1 protein. Journal of Animal and Plant Sciences.

[b24-tlsr-36-2-253] Livak KJ, Schmittgen TD (2001). Analysis of relative gene expression data using real-time quantitative PCR and the 2(−Delta Delta C(T)) method. Methods.

[b25-tlsr-36-2-253] Low L-Y, Ong Abdullah J, Wee C-Y, Sekeli R, Tan CK, Loh JY, Lai KS (2019). Effects of lignosulfonates on callus proliferation and shoot induction of recalcitrant *Indica* rice. Sains Malaysiana.

[b26-tlsr-36-2-253] Low L-Y, Yang S-K, Kok D, Ong-Abdullah J, Tan NP, Lai K-S, Çelik Ö (2018). Transgenic plants: Gene constructs, vector and transformation method. New visions in plant science.

[b27-tlsr-36-2-253] Meier SJ, Hatton TA, Wang DI (1999). Cell death from bursting bubbles: Role of cell attachment to rising bubbles in sparged reactors. Biotechnology and Bioengineering.

[b28-tlsr-36-2-253] Meng F, Xiang D, Zhu J, Li Y, Mao C (2019). Molecular mechanisms of root development in rice. Rice.

[b29-tlsr-36-2-253] Murashige T, Skoog F (1962). A revised medium for rapid growth and bio assays with tobacco tissue cultures. Physiologia Plantarum.

[b30-tlsr-36-2-253] Nishimura T, Hayashi K, Suzuki H, Gyohda A, Takaoka C, Sakaguchi Y, Matsumoto S, Kasahara H, Sakai T, Kato J, Kamiya Y, Koshiba T (2014). Yucasin is a potent inhibitor of YUCCA, a key enzyme in auxin biosynthesis. Plant Journal.

[b31-tlsr-36-2-253] Pan X, Welti R, Wang X (2010). Quantitative analysis of major plant hormones in crude plant extracts by high-performance liquid chromatography-mass spectrometry. Nature Protocols.

[b32-tlsr-36-2-253] Péret B, Swarup K, Ferguson A, Seth M, Yang Y, Dhondt S, James N, Casimiro I, Perry P, Syed A, Yang H, Reemmer J, Venison E, Howells C, Perez-Amador MA, Yun J, Alonso J, Beemster GT, Laplaze L, Murphy A, Bennett MJ, Nielsen E, Swarup R (2012). AUX/LAX genes encode a family of auxin influx transporters that perform distinct functions during Arabidopsis development. Plant Cell.

[b33-tlsr-36-2-253] Soeno K, Goda H, Ishii T, Ogura T, Tachikawa T, Sasaki E, Yoshida S, Fujioka S, Asami T, Shimada Y (2010). Auxin biosynthesis inhibitors, identified by a genomics-based approach, provide insights into auxin biosynthesis. Plant and Cell Physiology.

[b34-tlsr-36-2-253] Swarup R, Kramer EM, Perry P, Knox K, Leyser HM, Haseloff J, Beemster GT, Bhalerao R, Bennett MJ (2005). Root gravitropism requires lateral root cap and epidermal cells for transport and response to a mobile auxin signal. Nature Cell Biology.

[b35-tlsr-36-2-253] Terzi R, Kadioglu A, Kalaycioglu E, Saglam A (2014). Hydrogen peroxide pretreatment induces osmotic stress tolerance by influencing osmolyte and abscisic acid levels in maize leaves. Journal of Plant Interactions.

[b36-tlsr-36-2-253] Wiśniewska J, Kęsy J, Mucha N, Tyburski J (2024). Auxin resistant 1 gene (AUX1) mediates auxin effect on Arabidopsis thaliana callus growth by regulating its content and distribution pattern. Journal of Plant Physiology.

[b37-tlsr-36-2-253] Yu C, Sun C, Shen C, Wang S, Liu F, Liu Y, Chen Y, Li C, Qian Q, Aryal B, Geisler M, Jiang de A, Qi Y (2015). The auxin transporter, OsAUX1, is involved in primary root and root hair elongation and in Cd stress responses in rice (*Oryza sativa* L.). Plant Journal.

[b38-tlsr-36-2-253] Zhao Y, Cheng S, Song Y, Huang Y, Zhou S, Liu X, Zhou DX (2015). The interaction between rice ERF3 and WOX11 promotes crown root development by regulating gene expression involved in cytokinin signaling. Plant Cell.

[b39-tlsr-36-2-253] Zhao Y, Hu Y, Dai M, Huang L, Zhou DX (2009). The WUSCHEL-related homeobox gene WOX11 is required to activate shoot-borne crown root development in rice. Plant Cell.

